# VIP-2 —High-Sensitivity Tests on the Pauli Exclusion Principle for Electrons

**DOI:** 10.3390/e22111195

**Published:** 2020-10-22

**Authors:** Kristian Piscicchia, Johann Marton, Sergio Bartalucci, Massimiliano Bazzi, Sergio Bertolucci, Mario Bragadireanu, Michael Cargnelli, Alberto Clozza, Raffaele Del Grande, Luca De Paolis, Carlo Fiorini, Carlo Guaraldo, Mihail Iliescu, Matthias Laubenstein, Marco Miliucci, Edoardo Milotti, Fabrizio Napolitano, Andreas Pichler, Alessandro Scordo, Hexi Shi, Diana Laura Sirghi, Florin Sirghi, Laura Sperandio, Oton Vazquez Doce, Johann Zmeskal, Catalina Curceanu

**Affiliations:** 1 Centro Ricerche Enrico Fermi—Museo Storico della Fisica e Centro Studi e Ricerche “Enrico Fermi”, Piazza del Viminale 1, I-00184 Roma, Italy; kristian.piscicchia@lnf.infn.it; 2 INFN, Laboratori Nazionali di Frascati, Via E. Fermi 54, I-00044 Roma, Italy; Sergio.bartalucci@lnf.infn.it (S.B.); massimiliano.bazzi@lnf.infn.it (M.B.); bragadireanu.mario@lnf.infn.it (M.B.); alberto.clozza@lnf.infn.it (A.C.); Luca.DePaolis@lnf.infn.it (L.D.P.); guaraldo@lnf.infn.it (C.G.); mihai.iliescu@lnf.infn.it (M.I.); marco.miliucci@lnf.infn.it (M.M.); napolitano.fabrizio@lnf.infn.it (F.N.); scordo@lnf.infn.it (A.S.); sirghi@lnf.infn.it (D.L.S.); fsirghi@lnf.infn.it (F.S.); Laura.Sperandio@lnf.infn.it (L.S.); otonvazquezdoce@gmail.com (O.V.D.); Catalina.Curceanu@lnf.infn.it (C.C.); 3 Stefan Meyer Institute for Subatomic Physics, Kegelgasse 27, 1030 Vienna, Austria; michael.cargnelli@oeaw.ac.at (M.C.); shihexi@gmail.com (H.S.); Andreas.Pichler@oeaw.ac.at (A.P.); johann.zmeskal@oeaw.ac.at (J.Z.); 4 Dipartimento di Fisica e Astronomia, University of Bologna and INFN—Sezione di Bologna, Via Irnerio 46, I-40126 Bologna, Italy; Sergio.Bertolucci@lnf.infn.it; 5 Horia Hulubei National Institute of Physics and Nuclear Engineering, Str. Atomistilor No. 407, P.O. Box MG-6 Buchares-Magurele, Romania; 6 Excellence Cluster Universe, Technische Universität München, Boltzmannstraße 2, D-85748 Garching, Germany; 7 Politecnico di Milano, Dipartimento di Elettronica, Informazione e Bioingegneria and INFN Sezione di Milano, I-20133 Milano, Italy; carlo.fiorini@polimi.it; 8 INFN, Laboratori Nazionali del Gran Sasso, Via G. Acitelli 22, I-67100 L’Aquila, Italy; matthias.laubenstein@lngs.infn.it; 9 Dipartimento di Fisica, Università di Trieste and INFN—Sezione di Trieste, Via Valerio, 2, I-34127 Trieste, Italy; milotti@ts.infn.it

**Keywords:** Pauli Principle violation, X-ray spectroscopy, underground experiment

## Abstract

The VIP collaboration is performing high sensitivity tests of the Pauli Exclusion Principle for electrons in the extremely low cosmic background environment of the underground Gran Sasso National Laboratory INFN (Italy). In particular, the VIP-2 Open Systems experiment was conceived to put strong constraints on those Pauli Exclusion Principle violation models which respect the so-called Messiah–Greenberg superselection rule. The experimental technique consists of introducing a direct current in a copper conductor, and searching for the X-rays emission coming from a forbidden atomic transition from the L shell to the K shell of copper when the K shell is already occupied by two electrons. The analysis of the first three months of collected data (in 2018) is presented. The obtained result represents the best bound on the Pauli Exclusion Principle violation probability which fulfills the Messiah–Greenberg rule.

## 1. Introduction

The VIP collaboration is performing tests of the Pauli Exclusion Principle (PEP) for electrons, exploiting an experimental technique which searches for PEP-violating transitions, in various targets, using high precision X-rays detectors. PEP is a cornerstone of our theories of the microscopic world, which allows, for example, to calculate the Standard Model’s (SM) particles transition rates with extreme accuracy, and is responsible for many features in nature such as the stability of ordinary matter, astrophysical objects (e.g., neutron stars), and the existence of superconductivity.

Wolfgang Pauli introduced the PEP in order to explain the periodic table of the elements [1] and originally postulated that two identical fermions can not simultaneously occupy the same quantum state (see also Ref. [2]). In quantum mechanics, the Symmetrization Postulate (SP) generalizes the principle, asserting that the total wave function of a system of identical bosons/fermions is symmetric/antisymmetric with respect to their permutation [3,4]. Later on, the SP was demonstrated in the context of relativistic quantum field theory (RQFT) by Pauli himself, who gave a negative proof based on a series of group-theoretic and relativistic arguments [5]. Another proof was formulated by Lüders and Zumino [6] who showed that the Spin Statistics Theorem (SST) can be derived from few, very general assumptions (i.e., Lorentz/Poncaré and CPT symmetries, locality, unitarity, and causality) which are deeply embedded in the same structure of space and time. Theories beyond the SM, trying to unveil the fundamental connection between spin and statistics, or looking for an effective generalization which can accommodate tiny PEP violations, strongly need experimental verification. Ad-hoc experiments could shed new light on the fermionic/bosonic nature of the elementary particles, and on the very foundations of QFT.

The Messiah–Greenberg (MG) superselection rule [7] prevents transitions among states with different symmetry. Accordingly, a PEP-violating signal is to be searched in open quantum systems, i.e., by looking for transitions among violating states of a prepared system after the introduction of particles from outside of the considered system. Transitions among anomalous states would occur at the standard rate if the involved particles couple universally to the interaction field. The VIP-2 Open Systems experiment tests PEP violation (see e.g., Refs. [8,9,10,11,12,13,14,15,16,17,18,19]) constrained by the MG superselection rule. Recent calculations suggest that spin statistics violations emerge naturally in Quantum Gravity models (see Refs. [20,21,22]) which are not subject to the MG superselection rule. Consequently, they can be tested also by using closed systems as well, without the need to introduce test particles from outside in the system. An effort in this direction has been recently undertaken by the VIP collaboration with a new, dedicated set of experiments globally named VIP-2 Closed Systems.

**Figure 1:**
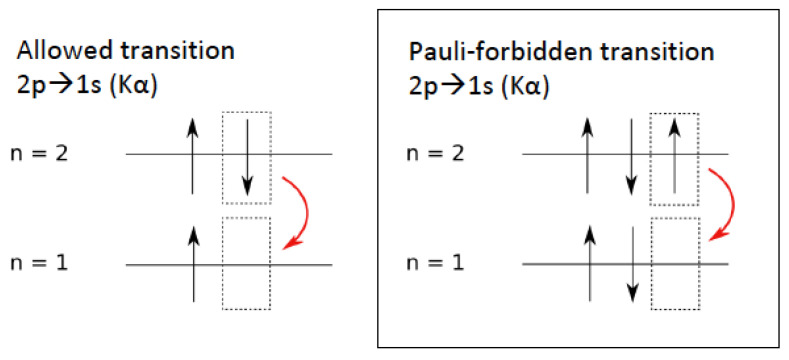
Schematic representation of a standard K${}_{\alpha }$ transition (**left**) and a Pauli Exclusion Principle (PEP)-violating transition (**right**).

The VIP-2 Open Systems experiment is operated in the extremely low-background environment of the underground Gran Sasso National Laboratory (LNGS) of INFN. The experimental method was suggested by Greenberg and Mohapatra [17] and a first experiment was performed by Ramberg and Snow [23]. The idea is to circulate a Direct Current (DC) in a copper strip conductor and look for anomalous K${}_{\alpha }$ transitions. An anomalous transition may occur if a new electron, injected into the copper strip, forms a wrong symmetry state with the electrons in the inner shell of a copper atom. The electron would be captured by the atom and emit anomalous X-rays as it cascades down to the fundamental level of the non-Paulian atom. The signature of a PEP violation would then be the detection of a PEP-violating $K_{\alpha }$ transition, which is a 2p→1s transition with the 1s level already occupied by two electrons (see Figure 1). The anomalous K${}_{\alpha }$ transition would be shifted by about 300 eV as a consequence of the extra shielding provided by the two electrons residing in the 1s state of the atom (see Refs. [24,25] for the details of the calculation). A reference background spectrum is collected with no circulating current.

In this paper, an upper limit on the PEP violation probability for electrons (which is usually quantified in terms of the parameter $\frac{1}{2}\beta^{2}$ [16,26]) is obtained from the analysis of the data collected by the VIP-2 Open Systems experiment in the data taking campaign of 2018. With respect to previous works (e.g., [23,27]) in which the expected number of electron–atom interactions was estimated in terms of scatterings in the conduction theory, in this analysis the limit is extracted on the base of a refined model. This is achieved accounting for the random walks performed by the diffusing and drifting electrons, and the random capture process, which may affect any of the electrons drifting and diffusing inside the target. We will show that such an improved interpretation of the data boosts the upper limit on the PEP violation probability by orders of magnitude.

## 2. Experimental Method of VIP-2

The aim of the VIP-2 Open Systems experiment is to greatly improve the previous result obtained by the VIP collaboration (see Ref. [27]). To this end, the experimental setup has been upgraded as follows:

- the VIP experiment used Charge Coupled Devices (CCDs) as X-ray detectors, which are characterized by a Full Width at Half Maximum (FWHM) of 320 eV at 8 keV. In order to improve the energy resolution at the anomalous transition energy 7746.73 eV (see Ref. [24]), the CCDs are replaced by Silicon Drift Detectors (SDDs) with a better energy resolution (190 eV FWHM at 8 keV) [28];
- the copper target is reshaped in order to increase the acceptance for the detection of the X-rays. The new target consists of two strips of copper (with a thickness of 50 μm, and a surface of 9 cm × 3 cm);
- the circulating DC current in the copper target is also increased, in order to enhance the pool of test electrons. To this end, a cooling pad (cooled down by a closed chiller circuit) is placed in between the two strips in order to avoid the temperature rise due to the heat dissipation in copper. This allows to increment the DC current from 40 A (in VIP) to 100 A;
- the timing resolution of the SDDs allows the implementation of a veto system which works as an active shielding, reducing the background originating from the high energy charged particles that are not shielded by the rocks of the Gran Sasso mountains. It is made of 32 plastic scintillators, each of size 250 mm × 38 mm × 40 mm, read from both sides by silicon photomultipliers.
- all the detectors and the front end preamplifier electronics are mounted inside the vacuum chamber which is kept at about 10^−6^ mbar during operation;
- the energy calibration and the measurement of the SDDs resolution is performed by means of a weakly radioactive Fe-55 source, with a 25 μm thick titanium foil attached on top, mounted together inside an aluminum holder. The fluorescence X-rays from titanium and manganese are used to calibrate the digitised channel into energy scale.

**Figure 2:**
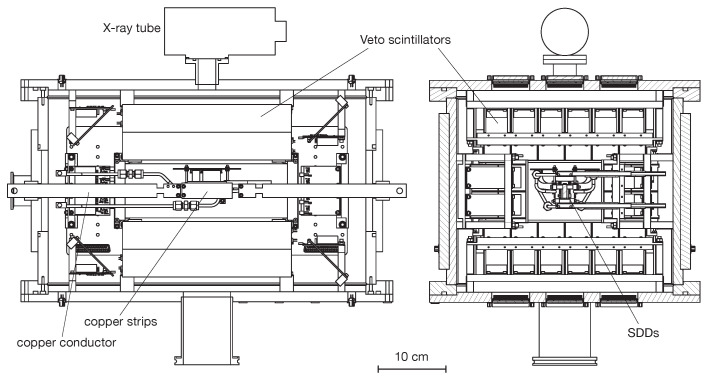
Side views of the design of the core components of the VIP-2 setup, including the Silicon Drift Detectors (SDDs) as the X-ray detector, the scintillators as active shielding with silicon photomultiplier readout.

The VIP-2 experimental apparatus was transported and mounted in the LNGS at the end of 2015. A first campaign of data-taking started in October 2016 and ended in November 2017. The detector system consisted of two arrays of 1 × 3 SDDs surrounding the copper target, each array with 3 cm^2^ of effective surface. The experimental setup was further upgraded in April 2018, when the detector system was replaced with two new arrays each one with 2 × 8 SDDs, for a total of 32 SDDs. The SDDs are cooled down to about −90 °C. The target system is cooled down by a water cooling keeping them to a temperature of about 12 degrees. When the current of 100 A is turned on, the strips’ temperature increases of few degrees and this induces a temperature rise at the SDDs of the same amount, which does not significantly alter the SDDs’ performances. A schematic view of the VIP-2 apparatus is shown in Figure 2.

**Figure 3:**
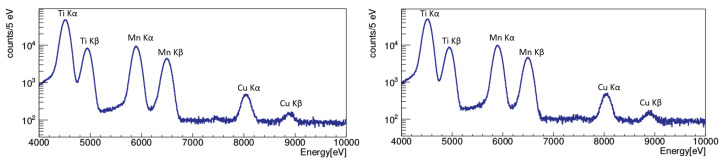
Energy calibrated spectra corresponding to about 42 days of data taking (during 2018) collected with current on (left), the spectrum collected with current off (right), which is normalized to the time of data taking with current on.

The energy calibrated spectra corresponding to a data collection period of about 42 days with current on (and about 42 days with current off), during 2018, are shown in Figure 3 (without current on the right, with current on the left, the current-off spectrum is normalized in time to the spectrum with current). The result of the data analysis, performed on this data set, is presented in Section 3.

In November 2018, the setup was surrounded with a passive shielding complex, composed of two layers of lead and copper blocks which will eliminate most of the background due to environmental gamma radiation. More details on the VIP-2 setup, the trigger logic, data acquisition, and slow control can be found in Ref. [24]. The VIP-2 Open Systems experimental setup is presently acquiring data in its final configuration.

## 3. Data Analysis

In the spectrum on the left in Figure 3, the fluorescence calibration lines of titanium and manganese are clearly recognizable; the K${}_{\alpha }$ and K${}_{\beta }$ transitions in copper are evident as well, together with a small excess at about 7.5 keV (corresponding to the nickel K${}_{\alpha }$ transition) due to the presence of nickel in the stainless steel components of the setup.

In the Region of Interest (ROI) ΔE=(7647–7847) eV, which corresponds to a FWHM window centered on the energy of the PEP violating transition, we do not observe any statistically significant excess. An upper limit on the PEP violation probability is then extrapolated, by calculating the maximum expected value of PEP violation signal counts, with a credibility level of 90%.

The total number of measured counts ($N_{wc}$)—with current on—in the ROI follows a Poisson distribution

(1) $$p\left(N_{wc}|\lambda_{wc}\right)=\frac{\lambda_{wc}^{N_{wc}}\cdot e^{-\lambda_{wc}}}{N_{wc}!}.$$

This random variable can be considered as given by the sum of a background and an eventual signal from PEP violating transitions $N_{wc}=N_{b}+N_{s}$, which are also Poissonian variables $N_{b}∼P_{\lambda_{b}}\;,\;N_{s}∼P_{\lambda_{s}}$ with expected values $\lambda_{b}$ and $\lambda_{s}$, respectively, satisfying the relation $\lambda_{wc}=\lambda_{b}+\lambda_{s}$. $N_{b}$ is the total number of measured counts—with current off—in the ROI. According to Bayes’ theorem, the posterior probability distribution function (pdf) of $\lambda_{s}$ is:

(2) $$p\left(\lambda_{s}|N_{wc},\lambda_{wc}\right)=\frac{p\left(N_{wc}|\lambda_{wc}\right)\cdot p_{0}\left(\lambda_{s}\right)}{\int_{0}^{\infty }p\left(N_{wc}|\lambda_{wc}\right)\cdot p_{0}\left(\lambda_{s}\right)d\lambda_{s}}.$$

For the prior $p_{0}\left(\lambda_{s}\right)$, we choose a Heaviside theta function

(3) $$p_{0}\left(\lambda_{s}\right)=\theta \left(\lambda_{s}^{max}-\lambda_{s}\right),$$

which, given the prior ignorance on $\lambda_{s}$, equally weights all non-negative values, up to the maximum value which is ruled out by previous experimental bounds; this is obtained from [24] and amounts to $\lambda_{s}^{max}=574$. As for what concerns $\lambda_{b}$, this is easily estimated. Since $N_{b}$ follows a Poisson distribution, from the Bayes theorem the pdf of $\lambda_{b}$ is a Gamma distribution function, with expectation value $\lambda_{b}=N_{b}+1$. From Equation (1) we then have:

(4) $$p\left(\lambda_{s}|N_{wc},\lambda_{wc}\right)=\frac{{\left(\lambda_{s}+\lambda_{b}\right)}^{N_{wc}}\cdot e^{-(\lambda_{s}+\lambda_{b})}\cdot \theta \left(\lambda_{s}-\lambda_{s}^{max}\right)}{\int_{0}^{\lambda_{s}^{max}}{\left(\lambda_{s}+\lambda_{b}\right)}^{N_{wc}}\cdot e^{-(\lambda_{s}+\lambda_{b})}\cdot d\lambda_{s}}.$$

The upper limit on the expected value of PEP violation signal counts (${\bar{\lambda }}_{s}$), corresponding to a credibility level of 90%, is then obtained by solving the following integral equation:

(5) $$\int_{0}^{{\bar{\lambda }}_{s}}p\left(\lambda_{s}|N_{wc},\lambda_{wc}\right)d\lambda_{s}=\frac{\Gamma \left(N_{wc}+1,\lambda_{b}\right)-\Gamma \left(N_{wc}+1,{\bar{\lambda }}_{s}+\lambda_{b}\right)}{\Gamma \left(N_{wc}+1,\lambda_{b}\right)-\Gamma \left(N_{wc}+1,\lambda_{s}^{max}+\lambda_{b}\right)}=0.9.$$

By substituting the values $N_{wc}=4103$ and $\lambda_{b}=4010$ in Equation (5) this yields ${\bar{\lambda }}_{s}=178$. The upper bound on the PEP violation probability is then given by:

(6) $$\frac{\beta^{2}}{2}\leq \frac{{\bar{\lambda }}_{s}}{N_{\mathrm{int}}N_{\mathrm{new}}\epsilon }\leq 4.5\times 10^{-42},$$

corresponding to a 90% credibility level. In Equation (6) $N_{\mathrm{new}}=\left(1/e\right)\int_{\Delta t}I\left(t\right)dt$ is the number of current electrons injected in the copper target over the data-acquisition time (with current) $\Delta t∼3.6\times 10^{6}$ s. As extensively described in Ref. [29], the electron–atom interaction time, which was used in the past (e.g., in Refs. [23,24]), deserves a revision based on a more accurate and realistic description of the electron motion inside the target. An interaction scheme which is based on the electron–atom scattering would yield an expected number of interactions $N_{\mathrm{int}}=D/\mu$, where *D* is the effective length of the copper strip and μ the scattering length for conduction electrons in the copper strip. However, scatterings in the conduction theory are mostly related to phonons and other lattice irregularities (see, e.g., Ref. [30]), and not to the capture process of electrons by atoms. In Ref. [29], this simplified approach to the electrons path was superseded, and a more accurate model was introduced which accounts for the diffusion of the electrons through the metal and their complex random walks, in a 1-D diffusion-transport context. The average time between close encounters is found to be $\tau_{E}∼3.3\times 10^{-17}$ s, while the traversal time of the copper target is estimated to amount to T∼15 s. This leads to a far larger number of electron–atom captures $N_{\mathrm{int}}=T/\tau_{E}$ with respect to the scattering scheme, thus greatly improving the limit on the PEP violation probability.

In order to account for the uncertainties which could affect the upper bound on $\beta^{2}/2$, the statistical and systematic effects related to all the parameters contained in the posterior distribution function of $\lambda_{s}$ are to be considered (see e.g., Ref. [31]). With this aim, we rewrite the pdf of $\lambda_{s}$ directly as a pdf of the PEP violation probability $\beta^{2}/2$ through the change of variable $\lambda_{s}\to \frac{\beta^{2}}{2}N_{int}N_{new}\epsilon$. The parameters which are affected by uncertainties are $\lambda_{b}$, $N_{int}$, $N_{new}$, ϵ, hence the pdf turns out to be

(7) $$\begin{array}{c} p\left(\beta^{2}/2|N_{wc}\right)=\int p\left(\beta^{2}/2|N_{wc},\lambda_{b},N_{int},N_{new},\epsilon \right)\cdot f_{1}\left(N_{int}\right)\cdot f_{2}\left(N_{new}\right)\cdot f_{3}\left(\epsilon \right)\cdot \\ \cdot f_{4}\left(\lambda_{b}\right)\cdot \;dN_{int}\;dN_{new}\;d\epsilon \;d\lambda_{b} \end{array}$$

where $f_{1}\left(N_{int}\right),f_{2}\left(N_{new}\right),f_{3}\left(\epsilon \right)$ and $f_{4}\left(\lambda_{b}\right)$ are the pdfs of $N_{int}$, $N_{new}$, ϵ and $\lambda_{b}$, respectively. For $f_{1}$, $f_{2}$, and $f_{3}$ we consider Gaussian distributions characterized by expectation values given by the nominal values of the parameters and standard deviations corresponding to their uncertainties (see Table 1).

According to the Bayes theorem, $f_{4}\left(\lambda_{b}\right)$ is a Gamma distribution:

(8) $$f_{4}\left(\lambda_{b}\right)=p\left(\lambda_{b}|N_{b}\right)=\frac{p\left(N_{b}|\lambda_{b}\right)\;p_{0}\left(\lambda_{b}\right)}{\int p\left(N_{b}|\lambda_{b}\right)\;p_{0}\left(\lambda_{b}\right)\;d\lambda_{b}}=\frac{\lambda_{b}^{N_{b}}\;e^{-\lambda_{b}}}{\int \lambda_{b}^{N_{b}}\;e^{-\lambda_{b}}\;d\lambda_{b}}\;,$$

under the assumption that the prior $p_{0}\left(\lambda_{b}\right)$ is uniform and $\lambda_{b}$ ranges over all possible finite values of the variable of interest. The upper limit on $\beta^{2}/2$, corresponding to a credibility level of 90%, is then obtained by solving the following integral equation:

(9) $$\int_{0}^{{\bar{\beta }}^{2}/2}p\left(\beta^{2}/2|N_{wc}\right)\;d\left(\beta^{2}/2\right)=0.9\;,$$

which yields

(10) $$\frac{\beta^{2}}{2}<5.4\times 10^{-42}\;.$$

## 4. Conclusions and Outlook

In this work, the outcome of the analysis of the first three months of collected data—during 2018—with the upgraded VIP-2 Open Systems setup was presented. The experiment is meant to test those models of PEP violation which are constrained by the Messiah–Greenberg superselection rule. The experiment sets the strongest bound on the Pauli Exclusion Principle violation probability for electrons at $\frac{1}{2}\beta^{2}<4.5\times 10^{-42}$. When the statistical and systematic uncertainties are also considered in the pdf of the PEP violation probability then the limit turns to be $\frac{1}{2}\beta^{2}<5.4\times 10^{-42}$. The Bayesian data analysis takes advantage of a new model [29], which, relying on a 1-D diffusion-transport calculation, accounts for the random walks, i.e., the diffusion and drift of the electrons when crossing the target. The analysis in Ref. [29] is mostly classical. We are presently working to extend the calculation to the quantum domain. We are also looking for possible interpretation of this data set in the framework of various models, including quantum gravity inspired ones.

## Figures and Tables

**Table 1:** Expectation values and standard deviations of all the parameters involved in the pdf of $\beta^{2}/2$.

$N_{\mathrm{int}}$	$N_{\mathrm{new}}$	ϵ
$\left(4.61\pm 0.09\right)\times 10^{17}$	$\left(2.25697\pm 0.00002\right)\times 10^{27}$	$\left(3.85\pm 0.05\right)\times 10^{-2}$

## Abbreviations

The following abbreviations are used in this manuscript:

VIP	Violation of Pauli
VIP-2	Violation of Pauli-2
INFN	Istituto Nazionale di Fisica Nucleare
PEP	Pauli Exclusion Principle
SP	Symmetrization Postulate
QFT	Quantum Field Theory
SST	Spin Statistic Theorem
MG	Messiah–Greenberg
LNGS	Gran Sasso National Laboratory
DC	Direct Current
CCD	Charge Coupled Devices
FWHM	Full Width at Half Maximum
SDD	Silicon Drift Detector
ROI	Region of Interest
pdf	probability distribution function
